# TUMOR NECROSIS FACTOR-Α, INTERLUKIN-6, AND VISFATIN LEVELS IN OLDER PATIENTS WITH MALNUTRITION

**DOI:** 10.1093/geroni/igad104.3675

**Published:** 2023-12-21

**Authors:** Hsien-Hao Huang

**Affiliations:** Taipei Veterans General Hospital, Taipei, Taipei, Taiwan (Republic of China)

## Abstract

**Introduction:**

Proinflammatory cytokine is associated with malnutrition status. Tumor necrosis factor-α (TNF-α) and interleukin-6 (IL-6) were pro-inflammatory cytokines stimulated by nutritional deficiency. Visfatin is an adipokine with a strong correlation with inflammation. The relationships of TNF-α, IL-6 and visfatin are not consistent, and no study have investigated them in the older patients with malnutrition.

**Methods:**

This prospective study included patients aged ≥ 75 years at the emergency department and all patients underwent Mini-Nutritional Assessment-Short Form (MNA-SF) and blood tests for fasting plasma TNF-α, IL-6 and visfatin levels.

**Results:**

We enrolled 106 older patients with a mean age of 87.3 years, including 43 (40.5%) patients in at risk of malnutrition group (MNA-SF between 8 to 11), 24 (22.6%) patients in the malnutrition group (MNA-SF < 8), and 39 (36.7%) patients in normal group. Compared to the normal group, both at risk of malnutrition and malnutrition group had significantly lower Mini-Mental State Examination (MMSE). Furthermore, TNF-α was significantly higher in at risk of malnutrition group. In contrast, IL-6 was significantly higher in malnutrition group. Visfatin levels was not correlated to the malnutrition status. Both TNF-α and IL-6 negatively correlated with Barthel index and MMSE. Backward and stepwise multiple logistic regression analyses showed that the independent predictor for both at risk of malnutrition and malnutrition was MMSE.

**Conclusion:**

The nutrition deficit status was correlated with proinflammatory cytokines, serum TNF-α and IL-6. The independent predictor of both at risk of malnutrition and malnutrition group was MMSE in older patients.

